# Immune response in the eye following epileptic seizures

**DOI:** 10.1186/s12974-016-0618-3

**Published:** 2016-06-27

**Authors:** Matilda Ahl, Una Avdic, Cecilia Skoug, Idrish Ali, Deepti Chugh, Ulrica Englund Johansson, Christine T Ekdahl

**Affiliations:** Inflammation and Stem Cell Therapy Group, Division of Clinical Neurophysiology, Lund University, BMC A11, Sölvegatan 17, SE-221 84 Lund, Sweden; Lund Epilepsy Center, Lund University, SE-221 85 Lund, Sweden; Division of Ophthalmology, Department of Clinical Sciences, Lund University, SE-221 85 Lund, Sweden

**Keywords:** Inflammation, Microglia, Astrocytes, Epilepsy, Retina, Brain

## Abstract

**Background:**

Epileptic seizures are associated with an immune response in the brain. However, it is not known whether it can extend to remote areas of the brain, such as the eyes. Hence, we investigated whether epileptic seizures induce inflammation in the retina.

**Methods:**

Adult rats underwent electrically induced temporal status epilepticus, and the eyes were studied 6 h, 1, and 7 weeks later with biochemical and immunohistochemical analyses. An additional group of animals received CX3CR1 antibody intracerebroventricularly for 6 weeks after status epilepticus.

**Results:**

Biochemical analyses and immunohistochemistry revealed no increased cell death and unaltered expression of several immune-related cytokines and chemokines as well as no microglial activation, 6 h post-status epilepticus compared to non-stimulated controls. At 1 week, again, retinal cytoarchitecture appeared normal and there was no cell death or micro- or macroglial reaction, apart from a small decrease in interleukin-10. However, at 7 weeks, even if the cytoarchitecture remained normal and no ongoing cell death was detected, the numbers of microglia were increased ipsi- and contralateral to the epileptic focus. The microglia remained within the synaptic layers but often in clusters and with more processes extending into the outer nuclear layer. Morphological analyses revealed a decrease in surveying and an increase in activated microglia. In addition, increased levels of the chemokine KC/GRO and cytokine interleukin-1β were found. Furthermore, macroglial activation was noted in the inner retina. No alterations in numbers of phagocytic cells, infiltrating macrophages, or vascular pericytes were observed. Post-synaptic density-95 cluster intensity was reduced in the outer nuclear layer, reflecting seizure-induced synaptic changes without disrupted cytoarchitecture in areas with increased microglial activation. The retinal gliosis was decreased by a CX3CR1 immune modulation known to reduce gliosis within epileptic foci, suggesting a common immunological reaction.

**Conclusions:**

Our results are the first evidence that epileptic seizures induce an immune response in the retina. It has a potential to become a novel non-invasive tool for detecting brain inflammation through the eyes.

## Background

Epilepsy is a neurological disorder characterized by spontaneous seizures, affecting almost 1 % of the population worldwide [1]. An epileptic seizure is an abrupt abnormal synchronized activity affecting the entire—or parts, of the brain. When a seizure engages the entire brain, called a generalized seizure, the patients experience disturbed consciousness and often tonic-clonic muscle movements. Seizures initiated in parts of the brain are named focal seizures and may induce a plethora of symptoms often correlated to the function or lack of function of that particular brain region. The etiology of epilepsy varies. It may arise due to a brain infection, trauma, ischemia, tumor, neurodegenerative disease, or genetic predisposition. A large proportion is cryptogenic. Almost 40 % of epilepsy patients are resistant to current anti-epileptic therapy, which makes investigation of new therapeutic targets and biomarkers for patient stratification warranted.

Pathological hallmarks in the brain associated with epilepsy include imbalance in synaptic transmission, neuronal damage, and an exaggerated immune response [2–6]. During the acute innate immune response in the brain following seizures, microglia release pro- and anti-inflammatory mediators [7], undergo phenotypic changes, and migrate towards the epileptic foci where they phagocytize cell debris [8, 9]. Moreover, astrocytes change their activity state and exhibit disturbed buffering capacity of ions and glutamate uptake [10]. In addition to the innate immune reaction, seizures may cause blood-brain barrier dysfunction and activation of vascular-associated and blood-derived immune cells [5]. Hitherto, a seizure-induced immune response has primarily been described within the epileptic foci of the brain, such as in hippocampal sclerosis in medial temporal lobe epilepsy, the most common form of epilepsy [4]. However, recent findings support the idea that seizures may be viewed as a disturbance of entire brain networks, including subcortical nodes for seizure propagation [11]. More remote areas of the brain, such as the retina, have not yet been investigated at all. Recently, we reported microglial activation in both cortical and subcortical brain areas even before behavioral seizures had developed in a genetic mouse model of epilepsy [12]. Therefore, the relationship between brain inflammation and epileptogenesis may also be further understood by studying the immune response in remote areas to the epileptic foci that are not known to either generate or propagate seizures.

In the present study, we therefore decided to explore whether a post-seizure immune response can extend beyond an epileptic focus within the temporal lobes and, hence, be detected as far as the retina of adult rats. The retina lines the back of the eyeball and constitutes a remote extension of the brain, so far not considered to exhibit seizure-induced pathology. It is a light-sensitive neural structure with several layers of strictly interconnected neurons [13]. The innate and adaptive immune response of the retina involves, similar to other brain regions, the activation of microglia and macroglial cells (i.e., astrocytes and Müller cells), vascular pericytes, and leucocytes that cross the blood-retina barrier (BRB) [14–16]. Since the retinal immune response is more accessible than the cerebral immune response for non-invasive investigations, it may become an attractive biomarker of brain inflammation if it reflects the immune response in the rest of the brain. Here, we evaluated micro- and macroglial activation, leukocyte infiltration, and number of vascular pericytes in the retina, acutely and late after electrically induced temporal status epilepticus. We also investigated whether a seizure-induced retinal immune response may be modulated by intracerebroventricular infusion of an antibody (Ab) against the chemokine receptor CX3CR1.

## Methods

### Animals

Adult male Sprague Dawley rats (*n* = 78) weighing between 200 and 250 g were procured from Charles River (Germany). The animals were housed in a 12-h light/dark cycle with ad libitum food and water. All experimental procedures followed the guidelines set by the Malmö-Lund Ethical Committee in Sweden for the use and care of laboratory animals, and the ARVO statement for the use of animals in ophthalmic and vision research.

### Group assignment

Animals were divided into three survival groups following electrically induced temporal status epilepticus (SE) and corresponding non-stimulated controls (NSC): 6 h (SE *n* = 4 and NSC *n* = 5 were perfused, SE *n* = 4 and NSC *n* = 4 were homogenized), 1 week (SE *n* = 9 and NSC *n* = 8 were perfused, SE *n* = 6 and NSC *n* = 6 were homogenized), and 7 weeks (SE *n* = 6, SE + CX3CR1 antibody *n* = 7 and NSC + saline infusion *n* = 9 were perfused SE *n* = 5 and NSC *n* = 5 were homogenized).

### Surgeries, drug infusions, and electrically induced temporal status epilepticus

**Fig. 1:**
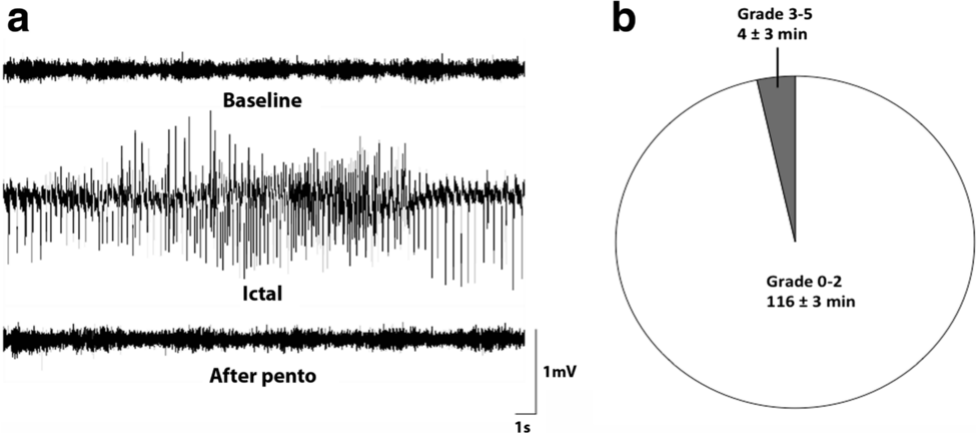
Electroencephalographic recordings from intrahippocampal electrodes before, during, and after electrically induced temporal status epilepticus (SE). **a** Representative baseline activity in rats before stimulations (*top*), high-frequency epileptiform activity during the 2 h of self-sustained SE (*middle*), and baseline activity after SE was terminated with pentobarbital injection (*bottom*). **b** Pie chart showing the relative distribution of different seizure semiology during the 2-h SE period. The semiology is based on the Racine’s scale [18], where stages 0–2 and 3–5 represent partial and generalized seizure semiology, respectively. Each segment depicts the mean percentage of time spent exhibiting the behavior

Animals were anesthetized with 2 % isofluorane and implanted with a bipolar-insulated stainless steel electrode (Plastics One, Roanoke, VA) into the right ventral CA1/CA3 region of the hippocampus (coordinates 4.8 mm posterior and 5.2 mm lateral from the bregma and 6.3 mm ventral from the dura, tooth bar set at −3.0 mm) for stimulation and recording. A unipolar electrode was placed between the skull and adjacent muscle to serve as ground electrode. In addition, a subset of animals were implanted with intracerebroventricular cannulae (Brain Infusion Kit 1, ALZET, USA): coordinates 1.0 mm posterior and 1.5 mm lateral to the bregma and 3.5 mm ventral to the flat skull position (with the bregma as reference) ipsilateral to the electrode placement for continuous saline or CX3CR1 antibody infusion (20 μg/ml; Abcam, UK) from a subcutaneously implanted osmotic pump (ALZET) during either 1 week after SE or during 6 weeks starting 1 week after SE. Following a week of recovery after surgery, rats were subjected to electrically induced temporal SE according to previously described protocol [17]. Electrode- and cannulae-implanted non-stimulated rats served as controls (NSC). Only rats that displayed self-sustained ictal electroencephalographic (EEG) activity for 2 h (Fig. 1) in the temporal lobe and mainly partial seizure semiology according to previous description [17], e.g., oralfacial twitches, nodding, drooling, and unilateral forelimb clonus, according to Racine’s scale, were included in this study [18]. Behavioral symptoms and ictal EEG activity were completely interrupted after 2 h of self-sustained SE by the administration of pentobarbital (65 mg/kg, intraperitoneal injection) (Fig. 1a).

### Tissue preparation

For biochemical analyses, rats were decapitated and the eyes, excluding the lenses, were immediately collected, frozen on dry ice, and stored in −80 °C. For immunohistochemistry, rats were perfused with ice-cold 0.9 % saline and paraformaldehyde (PFA) (4 % in 0.1 M phosphate-buffered saline (PBS), pH 7.4). The eyes were enucleated and post-fixed in PFA for 4 h, rinsed in PBS, incubated in 10 % sucrose (16 h) and 25 % sucrose (16 h), consecutively embedded in a Yazulla medium (30 % egg albumin, 3 % gelatin), and finally cut in 20 μm-thick sagittal cryosections (Microm HM 560, US) and stored at −20 °C.

### Multiplex enzyme-linked immunosorbent assay (ELISA)

Samples were homogenized on ice in buffer (pH 7.6) containing (in millimolars) 50.0 Tris-HCl, 150 NaCl, 5.0 CaCl_2_, 0.02 % NaN_3_, and 1 % Triton X-100, and then centrifuged at 17,000*g* for 30 min at 4 °C. The supernatant was collected into a microcentrifuge tube, where the total protein concentration was determined by BCA protein assay (BCA, Pierce, Rockford, IL) as per manufacturer’s instructions. Levels of interleukin (IL)-1β, tumor necrosis factor (TNF)-α, interferon (IFN)-γ, IL-4, IL-5, IL-6, IL-10, IL-13, and keratinocyte chemoattractant/growth-related oncogene (KC/GRO) were measured by sandwich immunoassay methods using commercially available electrochemiluminescent detection system, plates, and reagents (V-PLEX Proinflammatory Panel 2 (rat) kit, Meso Scale Discovery (MSD), Gaithersburg, MD, USA) as per manufacturer’s instructions with minor modifications. Briefly, 100 μg (50 μl) of the protein sample was loaded per well in the MSD plate. The samples were incubated overnight at 4 °C with shaking. For each assay, samples were analyzed in duplicates and compared with known concentrations of protein standards. Plates were analyzed using the SECTOR Imager 2400.

### Western blot analysis

Western blot analyses were performed as previously described [19]. The following primary Abs were used: mouse monoclonal anti-β actin (1:10,000; Sigma-Aldrich, MO, US), rabbit monoclonal anti-glyceraldehyde 3-phosphate dehydrogenase (GAPDH) (1:2000; Cell Signaling Technologies, CA, USA), rabbit polyclonal anti-CX3CR1 (1:500; Abcam, Cambridge, UK), and mouse monoclonal anti-postsynaptic density-95 (PSD-95) (1:200, Abcam). Secondary Abs used were either horseradish peroxidase-conjugated anti-mouse or anti-rabbit (both 1:5000; Sigma-Aldrich). Band intensities were quantified using ImageJ software (NIH, USA), and β-actin or GAPDH was used as a loading control.

### Fluoro-Jade staining

Sections were washed with potassium PBS, hydrated, and pretreated with 0.06 % potassium permanganate for 15 min, rinsed with distilled water, and treated with 0.001 % Fluoro-Jade (Histo-Chem, Jefferson, AR, USA) for 30 min. They were then washed with distilled water, dehydrated by treatment with ethanol and xylene, and coverslipped with PERTEX mounting medium.

### Immunohistochemistry and hematoxylin-eosin staining

Immunohistochemistry was performed as previously described [20]. The following primary Abs were used: rabbit polyclonal anti-Iba1 (1:1000; Wako, Japan), mouse anti-rat CD68/ED1 (1:200; AbD Serotec, NC, USA), rabbit anti-CD-45 (1:100; Santa Cruz Biotechnology, TX, USA), mouse anti-neuron glial antigen 2 (NG2) (1:200; Millipore, MA, USA), mouse anti-glial fibrillary acidic protein (GFAP) (1:400; Sigma-Aldrich), goat anti-Iba1 (1:250; AbD Serotec), mouse anti-PSD-95 (1:500; Abcam), rabbit anti- IL-6 (1:400; Abcam), rabbit anti-IL-4 (1:100, Abcam), and goat anti-IL-1β (1:100; Santa Cruz Biotechnology). Sections were incubated with appropriate primary Abs overnight at 4 °C and secondary antibody for 1 h at room temperature. For each immunohistochemical assessment, some eye sections went through the entire protocol without primary Abs incubation to serve as the negative controls. The following secondary Abs were used: Cy3-conjugated donkey anti-mouse/rabbit/goat (1:200; Jackson ImmunoResearch, UK), Alexa-488 conjugated donkey anti-mouse/rabbit (1:200; Invitrogen, NY, USA), and Cy2-conjugated donkey anti-rabbit (1:200; Jackson ImmunoResearch). For counterstaining of nuclei, the sections were coverslipped using 496-diamidino-2-phenylindole (DAPI)-containing VECTASHIELD mounting medium (Vector Laboratories, Burlingame, CA, USA) and stored in −20 °C until cell quantification. For gross morphological analyses, sections were stained with hematoxylin-eosin (Htx-eosin) for 1 min, dehydrated, and coverslipped using PERTEX mounting medium (HistoLab, Sweden).

### Morphological analyses, cell countings, and intensity measurements

First, an overall gross morphological analysis of retinal lamination was performed throughout the entire retina using light microscopy, in four sections from ipsi- and contralateral eyes, respectively. Second, detailed analyses were performed with regard to nuclear layer morphology using the ranking system 0–2 (0 = normal nuclear layer morphology and the presence of 0–10 pyknotic (shrunken) nuclei; 1 = islands of disseminated nuclear layers without nuclei (typically the size of 1–2 cells) and 11–20 pyknotic nuclei; 2 = completely disseminated nuclear layers and >20 pyknotic nuclei) as described previously [21].

**Fig. 2:**
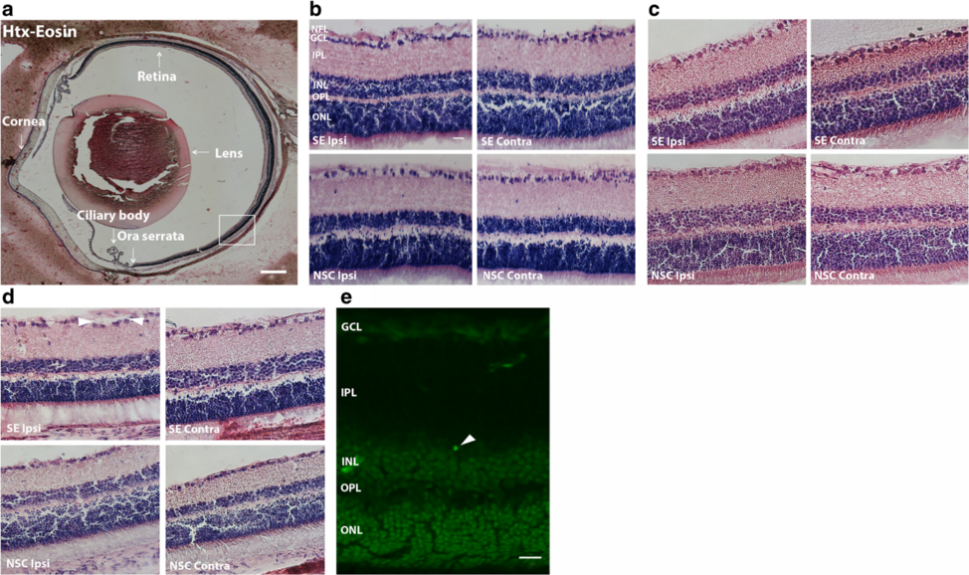
Lack of changes in cytoarchitecture in the retina after temporal SE. No changes in cytoarchitecture or gross morphology were found in the retina after SE. Representative photomicrographs representing the entire eye (**a**) and of gross morphology at 6 h (**b**), 1 week (**c**), and 7 weeks (**d**) following SE and in non-stimulated control rats (NSC) in ipsi- and contralateral retina related to seizure origin. *Arrow heads* (**d**) depict islands of disseminated GCL. Very few Flouro-Jade+ cells were found (**e**). *NFL* nerve fiber layer, *GCL* ganglion cell layer, *IPL* inner plexiform layer, *INL* inner nuclear layer, *OPL* outer plexiform layer, *ONL* outer nuclear layer. *Scale bars* are 500 μm in (**a**) and 50 μm for (**b**), (**c**), (**d**) and (**e**)

Quantifications of Iba1, ED1, CD45, and NG2-positive cells were performed in 8–12 regions of interest (ROIs) within 4–6 sections/eye, located in the peripheral retina, approximately 500 μm from the ora serrata (the junction between the retina and the ciliary body), using an Olympus BX61 epifluorescence microscope. The data is presented as the number of cells per ROI, which constituted an area of 16 × 10^4^ μm^2^ in the Iba1, ED1, NG2, and CD45 stainings. Each ROI included the following retinal layers: inner limiting membrane (ILM), nerve fiber layer (NFL), ganglion cell layer (GCL), inner plexiform layer (IPL), inner nuclear layer (INL), outer plexiform layer (OPL), and outer nuclear layer (ONL) (Fig. 2). For morphological analyses of microglia phenotypes in the same regions, a total number of 240 (6 h) and 120 (1 and 7 weeks) Iba1^+^ cells were analyzed per eye for three different subtypes: ramified (small soma and extensive dendritic tree), intermediate (larger soma and less extensive dendritic tree), and round/amoeboid (no processes), according to previously described definitions [12, 19]. The relative occurrence of each subtype was expressed as the mean percentage of Iba1^+^ cells per eye. The morphological assessments were further confirmed by quantifications of the length of the most extended process per Iba1^+^ cell, and Iba1^+^ cell soma diameter in ten Iba1^+^ cells per retina, from the same ROIs in the peripheral ipsilateral retina as the morphological evaluations, using Cell Sense Olympus software and Olympus BX61 epifluorescence microscope.

Semi-quantitative analysis of GFAP expression was performed in 12 representative images from four sections/eye, using Olympus BX61 epifluorescence microscope. The density of GFAP^+^ radial Müller cell processes was graded according to a 0–5 scale in IPL (0 = none, 1 = 1–5, 2 = 6–10, 3 = 11–20, 4 = 21–50, 5 = >50 processes) and a 0–3 scale in INL + OPL and ONL, respectively (0 = none, 1 = 1–5, 2 = 6–10, 3 = >10 processes). In addition, number of GFAP^+^ retinal astrocytes was manually quantified in 12 representative images of the inner retina from four sections/eye.

Intensity measurements of GFAP expression in the GCL, NFL, and ILM were performed in fluorescence images from 12 sections/eye (each image corresponding to 13 × 10^3^ μm^2^), using an Olympus BX61 epifluorescence microscope (Leica, Germany). Intensity measurements of PSD-95 expression in IPL, INL, OPL, and ONL, respectively, were performed with confocal laser scanning microscope (Zeiss, Germany) with a 561-nm excitation filter, ×63 oil-immersion objective, and ×5 zoom. Intensity measurements of IL-6, IL-4, and IL-1β expression in IPL, INL, OPL, and ONL, respectively, were carried out with a 488-nm and 561-nm excitation filter, ×40 oil-immersion objective, and ×5 digital zoom. Images were taken from three representative areas from each animal. Each image was acquired in a *z*-stack at an interval of 0.2 μm, on average 50 slices per z-stack. The images were analyzed in ImageJ software and the brightness and contrast corrected and noise reduced using the built-in ImageJ functions. Background intensity was measured in every image and subsequently subtracted from the mean gray value from each image in order to obtain a background-corrected mean gray value per animal.

To ensure the lack of bias, all analyses were conducted by an observer blind to the treatment conditions.

### Statistics

Statistical analyses were performed with the unpaired Student’s *t* test when comparing two groups, using GraphPad Prism software, except for analyses of gross morphology and grading of GFAP^+^ processes where Mann-Whitney’s rank sum test for non-parametric variables was applied. Microglial morphology was analyzed with two-way analysis of variance (ANOVA) followed by a Bonferroni post hoc test. Data are presented as means ± SEM, apart from gross morphology and GFAP^+^ process evaluations, which are presented as median ± range with upper quartile range. Differences were considered statistically significant at *p* ≤ 0.05.

## Results

### Lack of acute changes in the expression of immune mediators, glial activation, and cell death in the eyes after temporal status epilepticus

**Fig. 3:**
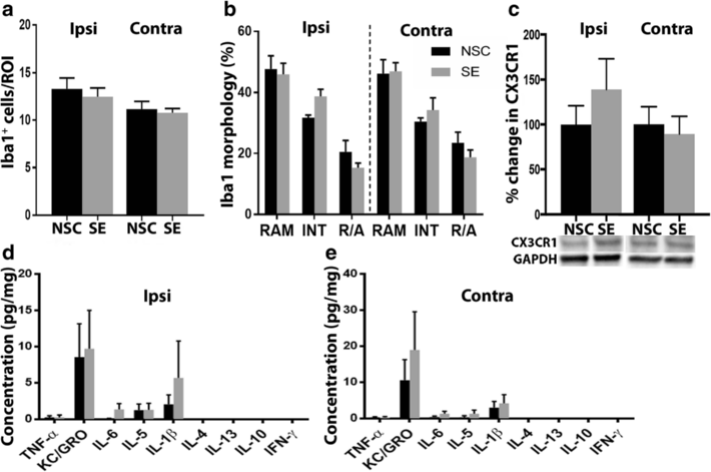
No changes in microglial activation or cytokine and chemokine protein expression in the eyes 6 h after temporal SE. No changes were observed in number of Iba1+ cells (**a**) or in Iba1+ cell morphology (**b**) between NSC and SE. Representative Western blot and quantification of CX3CR1 protein (~50 kDa) relative to NSC in the ipsilateral and contralateral eye 6 h post SE (**c**). Quantification of cytokine and chemokine protein expression, using mesoscale multiplex ELISA, in ipsilateral (**d**) and contralateral (**e**) eye homogenates 6 h after SE showed no differences compared to NSC. Data are presented as mean ± SEM, *n* = 5 NSC and *n* = 4 SE for WB and ELISA, *n* = 4 NSC and *n* = 4 SE for cell count and morphology. *p ≤ 0.05, unpaired *t* test in (**a**) and (**c**–**e**) two-way ANOVA in (**b**)

We performed cytoarchitectural analyses of the eye tissue. However, in the retina at the time point 6 h post-SE, no obvious pathological signs were found at the gross cytoarchitectural level, when assessed using Htx-eosin staining (Fig. 2a), with the retina showing well-defined and homogenous nuclear and synaptic layers and nerve fiber layer. Detailed morphological analysis of the retina revealed no differences between SE and NSC group in the ipsi- and contralateral eyes, with both retinas displaying well-preserved nuclear layer morphology and no pyknotic cells and disseminated GCL at 6 h (ipsilateral SE 0 *u* = 0 vs NSC 0 *u* = 0 and contralateral SE 0 *u* = 0 vs NSC 0 *u* = 0; Fig. 2b). Less than three Fluoro-Jade^+^ cells were observed in total per animal in both SE and NSC groups (Fig. 2e), which made statistical analyses inadequate. In order to detect an acute immune response in the eye, we first performed immunohistochemical evaluations of number of Iba1^+^ cells and the morphology of the microglia, including ramified, intermediate, and round/amoeboid phenotypes. However, no differences in Iba1^+^ cell number (Fig. 3a) or microglial morphology (Fig. 3b) could be detected. Seizure-induced microglial activation is also associated with a more phagocytic profile [6] and we therefore double-stained Iba1^+^ cells with the phagocytic marker ED1. Though, the number of Iba1^+^/ED1^+^ cells was too low to be adequately analyzed (rarely more than 1–2 cells per retina in both NSC and SE group).

Next, biochemical analyses of pro- and anti-inflammatory mediators in the protein homogenates from the eye tissue excluding the lens at 6 h following SE were evaluated. At this time point, several immune factors are upregulated in the epileptic focus of the hippocampus (Avdic U, Ahl M, Ekdahl CT, unpublished observation). However, even though the chemokine KC/GRO, chemokine receptor CX3CR1, and the cytokines IL-6, IL-5, and IL-1β were detectable in the eyes, the levels were unaltered compared to the NSC group, both ipsi- and contralateral to the epileptic focus (Fig. 3d, e).

### Lack of subacute changes in the expression of immune mediators, glial activation, and cell death in the eyes after temporal status epilepticus

We next performed cytoarchitectural analyses of the eye tissue 1 week post-SE. At this time point, neuronal necrosis and apoptotic cell death are readily observed in varying degree within temporal structures, including the hippocampus [17, 22]. However, in the retina, again, no obvious pathological signs were found at the gross cytoarchitectural level. Detailed morphological analysis of the retina showed no differences between SE and NSC group in the ipsi- and contralateral eyes, with both retinas displaying well-preserved nuclear layer morphology and only occasional pyknotic cells and disseminated GCL (ipsilateral SE 0.25 *u* = 0.875 vs NSC 0.5 *u* = 0.5 and contralateral SE 0 *u* = 0.75 vs NSC 0.25 *u* = 0.458 score) (Fig. 2c). Less than three Fluoro-Jade^+^ cells per animal were observed in both groups.

**Fig. 4:**
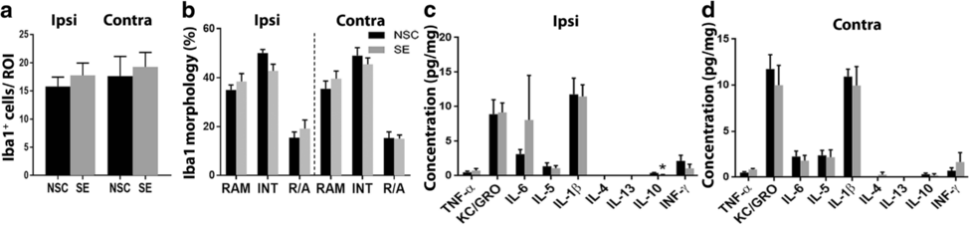
No changes in microglial activation or cytokine and chemokine protein expression in the eyes 1 week after temporal SE. Number of Iba1^+^ cells (**a**) and the Iba1^+^ cells morphology (**b**) did not differ between NSC and SE. Biochemical analyses of cytokine and chemokine protein expression in the ipsilateral (**c**) and contralateral (**d**) eye detected a small decrease in IL-10 in the ipsilateral eye. Data are presented as mean ± SEM, *n* = 6 NSC and *n* = 6 SE for ELISA and *n* = 8 NSC and *n* = 9 SE for cell count and morphology. *p ≤ 0.05, unpaired *t* test in (**a**) and (**c**–**d**), two-way ANOVA in (**b**)

Furthermore, no differences in Iba1^+^ cell number (Fig. 4a) or microglial morphology (Fig. 4b) could be detected. The number of Iba1^+^/ED1^+^ cells was less than 2–3 cells per retina in both groups. The expression of immune mediators was unaltered, apart from a small decrease in IL-10 in the ipsilateral eye (Fig. 4c, d).

### Delayed glial activation in the retina after temporal status epilepticus

We hypothesized that a seizure-induced tissue injury may be delayed in the retina compared to other brain structures and, therefore, we extended our analyses to a later time point, i.e., 7 weeks after SE. Again, no changes in retinal laminar organization or significant changes in the cytoarchitecture (ipsilateral SE 1.0 *u* = 1 vs NSC 0.5 *u* = 0.75 and contralateral SE 0.25 *u* = 0.375 vs NSC 0.333 *u* = 0.50 score) could be detected (Fig. 2d). The quantification of Fluoro-Jade^+^ cells in the retina never exceeded three cells per eye.

**Fig. 5:**
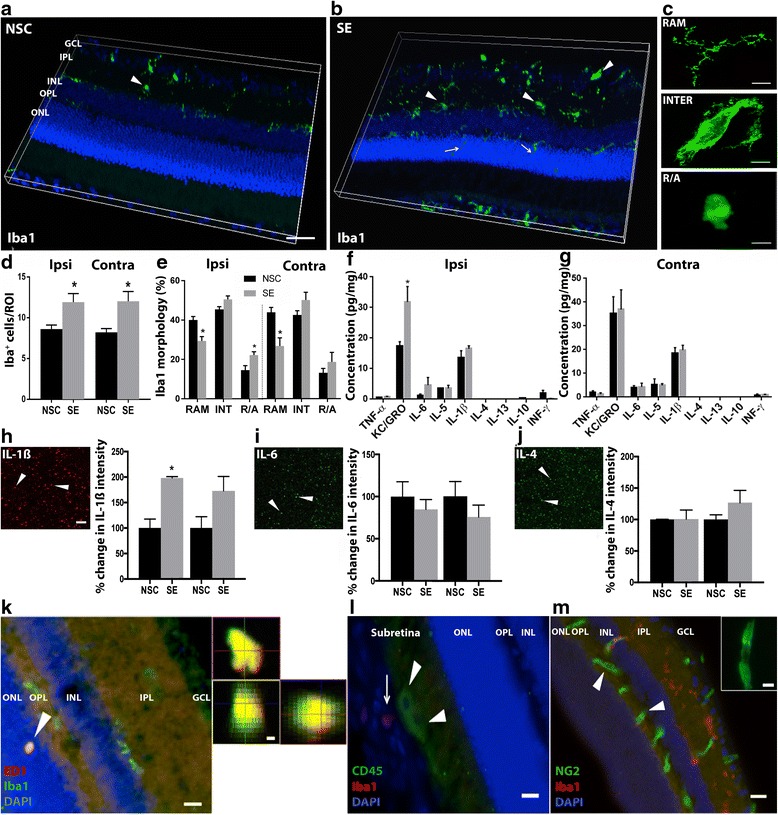
Delayed microglial activation in the retina 7 weeks after temporal SE. Representative confocal photomicrographs of microglial activation in non-stimulated controls NSC (**a**) and 7 weeks after SE (**b**). *Arrow heads* in (**a**) and (**b**) depict Iba1^+^ cells in IPL and GCL. *Arrows* in **B** mark Iba1^+^ processes in the ONL. Representative images of different Iba1^+^ cell morphologies, including ramified (RAM), intermediate (INT), and round/amoeboid (R/A) (**c**). Note the elongated cell soma and thicker proximal processes in INT compared the RAM cells. Quantification of numbers of Iba1^+^ cells in the ipsi- and contralateral retina 7 weeks following SE compared to NSC showed an increase after SE (**d**). Quantification of the relative percentage of microglia with the three different morphologies revealed a relative reduction in ramified and an increase in amoeboid morphology in the SE group (**e**). Biochemical analysis detected a SE-induced increase in chemokine KC/GRO levels in ipsilateral retina (**f**), but no changes in the contralateral eye (**g**). Representative pictures and intensity measurements in the IPL of cytokine IL-1β (**h**), IL-6 (**i**), and IL-4 (**j**) immune staining showed increased levels of IL-1β only. Confocal images of Iba1 and ED1 immunostaining of the retina (*left*) and orthogonal projection of an Iba1^+^ ED1^+^ cell (*right*) (**k**), Iba1 and CD45 immunostaining of the retina (**l**), and Iba1 and NG2 immunostaining of the retina with NG2^+^ cells in higher magnification in *inset* (**m**). Data are presented as means ± SEM, *n* = 5 NSC and *n* = 5 SE for ELISA, *n* = 9 NSC and *n* = 6 SE for cell quantification and evaluation. *p ≤ 0.05 un-paired *t* test in (**d**) and (**i**–**j**), 2-way ANOVA in (**e**). Scale bars are 500 μm for (**a**) and (**b**), 10 μm for (**c**) 5 μm for (**h**-**j**), 25 μm for (**k**–**m**), and insets 3 μm for (**k**) and 5 μm for (**m**)

The immune response in epileptic foci is often characterized by a prominent activation of microglial cells, which can be detected both acutely and chronically after SE [6, 23]. In the retina of both the NSC and 7 weeks post-SE group, the Iba1^+^ microglial cells were primarily located in the synaptic layers (OPL and IPL) and the GCL (Fig. 5a, b). The morphology of the microglia included again ramified, intermediate, and round/amoeboid phenotypes (Fig. 5c). Interestingly, the number of Iba1^+^ cells was increased bilaterally (Fig. 5d), with a few aberrant cell clusters in the INL and the occurrence of processes in the ONL (Fig. 5b). Accordingly, the morphology of the Iba1^+^ cells significantly changed. The SE group showed a relative decrease in ramified and an increase in round/amoeboid microglial phenotype with a significant interaction between groups and morphology in both the ipsi- and contralateral retina (Fig. 5e). The morphological differences were further confirmed in a subset of animals (*n* = 3 + 3, NSC and 7 weeks post-SE group, respectively) with larger Iba1^+^ cell soma diameter (SE 13.59 ± 0.71 vs NSC 9.57 ± 0.27 μm) and less extended processes per Iba1^+^ cell (SE 28.18 ± 2.11 vs NSC 38.78 ± 2.73 μm) in the ipsilateral retina of SE rats. Biochemical analyses of eye homogenates showed increased KC/GRO levels in the ipsilateral eye (Fig. 5f, g). Intensity measurement of immonohistochemical stainings for three pro- and anti-inflammatory markers, showed regional alterations in Il-1β levels, with an increase in IPL (Fig. 5h) in the ipsilateral eye, while the intensity of IL-6 or IL-4 remained unaltered (Fig. 5i, j). No changes in Il-1β, IL-6, and IL-4 intensity could be detected in other retinal layers including INL, OPL, and ONL (data not shown).

Less than 1–2 single-labeled ED1^+^ cells and Iba1^+^/ED1^+^ cells per retina were detected at 7 weeks post-SE (data not shown). The cells were uniformly distributed among the retinal layers (Fig. 5k). In order to evaluate a possible systemic contribution of leukocytes to the immune response in the retina after SE, numbers of CD45^+^ cells were evaluated at 7 weeks, but again, very low cell numbers were found with no differences between the two groups (ipsilateral SE 0.38 ± 0.13 vs NSC 0.97 ± 0.22, contralateral SE 0.88 ± 0.38 vs NSC 1.53 ± 0.34). At both 1 and 7 weeks, CD45^+^ cells were primarily located in the subretinal layers, not overlapping with the Iba1 staining (Fig. 5l).

In an attempt to define whether the immune response in the retina was associated with a subtle microvascular disturbance, we also analyzed the numbers of vascular NG2-expressing pericytes [16]. NG2^+^ cells were found in the same retinal layers as the Iba1^+^ cells, but their expressions did not overlap (Fig. 5m). Often, the NG2^+^ cells were aligned in a cluster of 2–3 cells, as if embracing a microvessel (Fig. 5m inset). However, the cell numbers did not differ between SE and NSC group neither 1 nor 7 weeks post-SE (1 week ipsilateral SE 9.64 ± 0.40 vs NSC 8.76 ± 0.62 and contralateral SE 8.67 ± 0.29 vs NSC 9.60 ± 0.51; 7 weeks ipsilateral SE 7.97 ± 0.64 vs NSC 8.38 ± 0.31, contralateral SE 7.47 ± 0.51 vs NSC 8.16 ± 0.76).

**Fig. 6:**
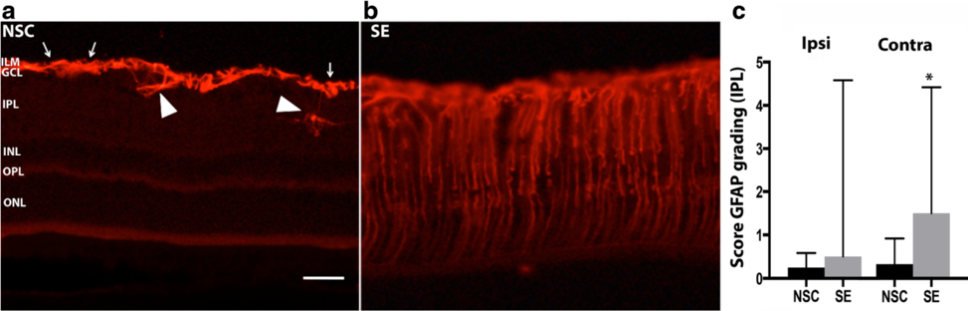
Delayed macroglial activation in the retina after temporal SE. Representative photomicrographs of GFAP expression on macroglia in the retina of NSC (**a**) and 7 weeks post-SE (**b**). The extension of GFAP^+^ processes and their end feet in different retinal layers are *visualized in* (**b**). *Arrows* depict GFAP^+^ end feet of Müller cells in ILM and *arrow heads* mark GFAP^+^ retinal astrocytes. Quantification of GFAP^+^ Müller cell processes in the IPL showed increased expression after SE (**c**). Data are presented as median range with upper quartile range, *n* = 9 NSC and *n* = 6 SE group (**c**). *p ≤ 0.05 Mann-Whitney’s rank sum test. Scale bar is 50 μm for (**a**) and (**b**)

Apart from microglial activation, seizure-induced gliosis in the brain is also associated with a strong astrocytic reaction, which is readily evaluated by the typical upregulation of GFAP [24, 25]. In the retina, Müller cells and retinal astrocytes, together referred to as macroglia, are responsible for injury-induced gliosis [26]. In NSC rats at 7 weeks post-SE, only weakly labeled GFAP^+^ Müller cell end feet/processes and astrocytes were found in ILM and GCL (Fig. 6a, arrows). We graded the number of GFAP^+^ Müller cell processes in the IPL, INL + OPL, and ONL, respectively, and found extensive GFAP^+^ staining in the ILM and GCL and, occasionally, also in the Müller cell end feet in the outer limiting membrane outside the ONL. It was significantly increased in the IPL contralaterally and there was a trend towards an increase (*p* = 0.07) ipsilaterally to the epileptic focus (Fig. 6a, b). The increase was also significant in the INL and OPL on the contralateral side (ipsilateral SE 0 *u* = 1.56 vs NSC 0 *u* = 0, contralateral SE 0.13 *u* = 1.04 vs NSC 0 *u* = 0). No differences were found in the ONL (data not shown). The high variation in glial activation in the SE group did not correlate to SE severity (total time a rat exhibited generalized tonic-clonic seizures during the 2 h of SE (regression analysis *p* = 0.7, *r*-value = −0.12), as previously seen for microglial activation in the brain [6]). This increase in number of GFAP^+^ processes at 7 weeks was not evident at either 6 h or 1 week post-SE (data not shown). However, at these acute and subacute time points, high variation within both the NSC and SE group made statistical analyses uncertain. At 6 h post-SE, we even detected a small decrease in GFAP^+^ processes in the IPL contralateral to the epileptic focus (ipsilateral SE 1.083 *u* = 1.21 vs NSC 1.694 *u* = 2.93, contralateral SE 1 *u* = 1.04 vs NSC 1.74 *u* = 3.48) compared to NSC. In addition, the overall intensity of GFAP immunostaining was measured in the ILM, NFL, and GCL but showed no differences either 6 h, 1, or 7 weeks post-SE compared to NSC group (6 h ipsilateral SE 19.6 ± 3.3 vs NSC 16.7 ± 2.7, contralateral SE 23.5 ± 2.7 vs NSC 21.5 ± 1.0; 1 week ipsilateral SE 26.94 ± 2.10 vs NSC 24.31 ± 2.05, contralateral SE 24.62 ± 2.70 vs NSC 25.48 ± 2.06; 7 weeks ipsilateral SE 18.61 ± 1.53 vs NSC 19.09 ± 0.89, contralateral SE 19.93 ± 1.62 vs NSC 18.73 ± 0.60). At all time points, in both the NSC and SE group, small cell bodies of GFAP^+^ retinal astrocytes with relatively thin processes were found also in the IPL (Fig. 6a, arrow heads). However, the number of GFAP^+^ retinal astrocytes in the IPL did not differ at 7 weeks (ipsilateral SE 0.14 ± 0.06 vs NSC 0.07 ± 0.06, contralateral SE 0.03 ± 0.02 vs NSC 0.05 ± 0.03).

### Altered synaptic protein expression in the retina after temporal status epilepticus

**Fig. 7:**
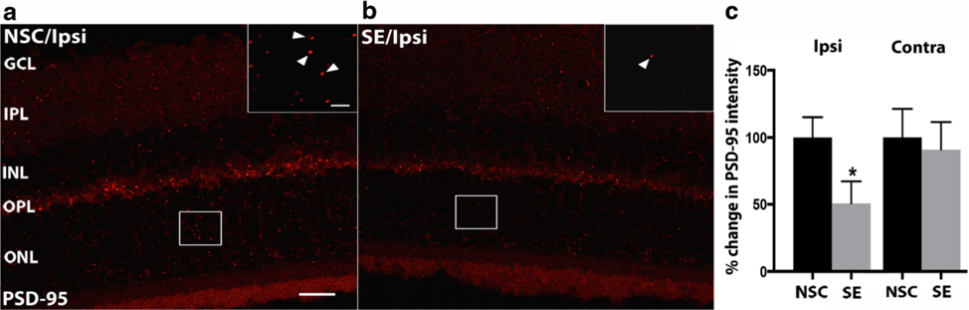
Reduced PSD-95 expression after temporal SE. Representative confocal photomicrographs of PSD-95 scaffolding protein clusters (*arrow heads* in *inset*) in the retina of NSC (**a**) and 7 weeks post-SE (**b**). Quantification of intensity measurements relative to NSC of PSD-95 clusters in ONL of ipsi- and contralateral retina 7 weeks post-SE compared to NSC showed reduced levels after SE in the ipsilateral retina (**c**). Data are presented as means ± SEM, *n* = 9 NSC and *n* = 6 SE group. *p ≤ 0.05 un-paired *t* test compared to controls. Scale bars are 50 μm for (**a**) and (**b**), and 5 μm in *inset*

Even if the epileptic seizures did not induce a prominent cell loss with changes in the cytoarchitecture of the retina, it may still induce a more subtle imbalance in synaptic transmission between neurons. The activation of micro- and macroglia may indicate such an ongoing synaptic/neuronal dysfunction. We have previously shown that levels of the scaffolding protein PSD-95, expressed primarily in excitatory synapses, may change due to an immune response in the brain [20]. Here, we measured the protein levels of PSD-95 in the eye 6 h post-SE but found no differences (ipsilateral SE 135.40 ± 44.07 *n* = 3 vs NSC 100.00 ± 50.44 *n* = 4, contralateral SE 172.20 ± 57.34 *n* = 3 vs NSC 100.00 ± 27.92 *n* = 5). However, intensity measurements of PSD-95 clusters 1 and 7 weeks post-SE in the different retinal layers revealed a small decrease in the ONL at 7 weeks in the retina ipsilateral to the epileptic focus (Fig. 7a–c).

### Intracerebroventricular CX3CR1 antibody infusion decreases micro- and macroglial activation in the retina after temporal status epilepticus

**Fig. 8:**
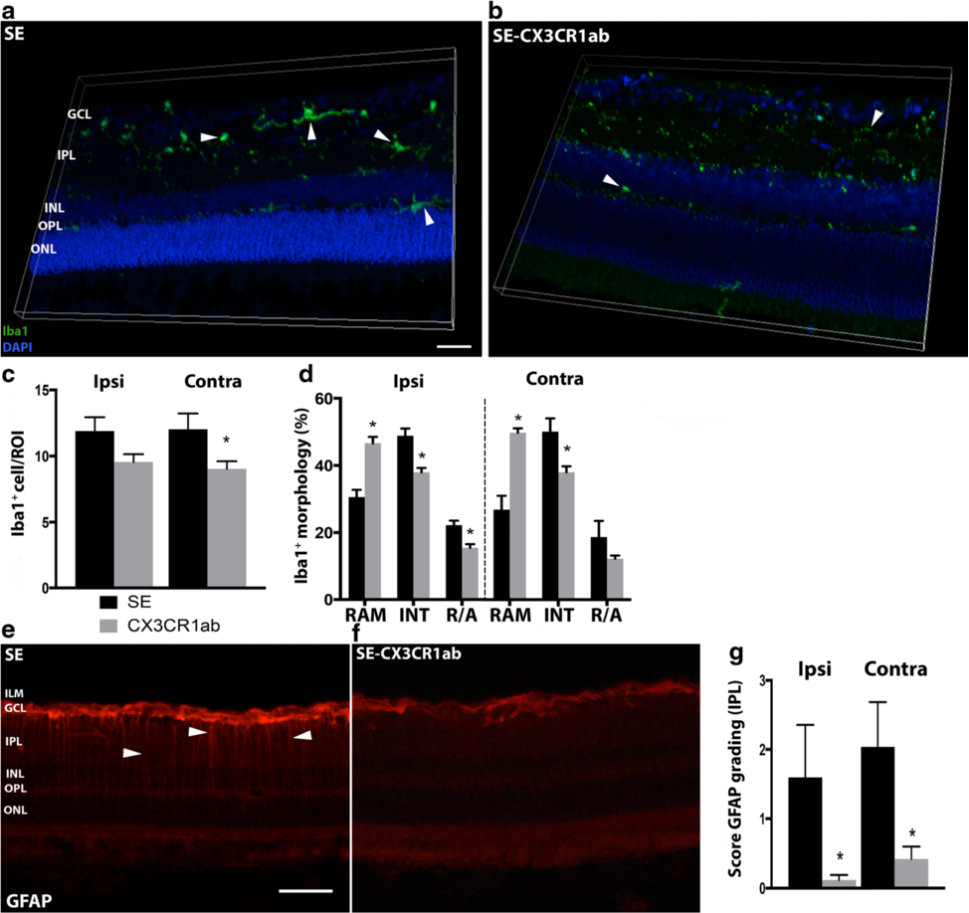
Decreased seizure-induced glial activation in the retina after CX3CR1 antibody treatment. Representative photomicrographs of the retina 7 weeks after SE (**a**) and CX3CR1 antibody-treated SE (**b**). *Arrow heads* depict Iba1^+^ cells in the IPL and OPL. Quantification of numbers of Iba1^+^cells in the ipsi- and contralateral retina at 7 weeks revealed a decrease after CX3CR1 treatment (**c**). Quantification of the relative percentage of microglia with different morphologies at 7 weeks showed a relative increase in ramified and a reduction in intermediate and amoeboid morphologies (**d**). Representative images of GFAP expression in macroglia in the retina 7 weeks post-SE (**e**) and post-SE with CX3CR1-treatment (**f**). GFAP^+^ processes in the IPL are marked with *arrow heads* in (**e**). Quantification of GFAP^+^ Müller cell processes in the IPL showed reduced numbers after SE (**g**). Data are presented as means ± SEM in (**c**) and (**d**), and as median range with upper quartile range in (**g**), *n* = 6 SE and *n* = 7 CX3CR1-treated SE group. *p ≤ 0.05 un-paired *t* test in (**c**), 2-way ANOVA in (**d**), Mann-Whitney’s rank sum test in (**g**). †*p* = 0.067 in (**c**). Scale bars are 500 μm for (**a**) and (**b**), and 50 μm for (**e**) and (**f**)

In order to evaluate possible similarities between the immune reaction in the retina and within the epileptic focus, an antibody (Ab) against the chemokine receptor CX3CR1 was infused intracerebroventricularly during 6 weeks starting 1 week after SE. The CX3CR1 is expressed on the microglia in the brain, including the retina, and can be upregulated both before and after seizures. Its ligand, fractalkine, is expressed by neurons and glial cells [27, 28]. Recently, we showed that inhibition of CX3CR1 with the same CX3CR1 Ab decreases microglial activation within the temporal epileptic foci [19]. Then, micro- and macroglial activation was evaluated in the retina 7 weeks post-SE. We observed an almost 25 % significant decrease in the number of Iba1^+^ cells in the contralateral retina in the SE group treated with CX3CR1 Ab, compared to the SE group with saline infusion, and there was a trend towards a decrease (*p* = 0.07) in the ipsilateral retina (Fig. 8a–c). The morphology of Iba1^+^ cells was also changed with a higher percentage of ramified and less intermediate and round phenotypes in the CX3CR1 Ab-treated SE group on both the ipsi- and contralateral side (Fig. 8d). In addition, the number of GFAP^+^ Müller cell processes was decreased in the CX3CR1 Ab-treated group, with fewer processes in the IPL in both ipsilateral and contralateral retina (Fig. 8e–g). However, the decrease did not reach significance in the INL and OPL on either sides (data not shown). The decrease in the IPL was not due to the differences in seizure severity during the SE (data not shown).

## Discussion

Here, we provide the first evidence that epileptic seizures arising within the temporal lobes of the adult rat brain lead to glial activation in the retina. The activation includes both micro- and macroglia and is delayed in the retina compared to the epileptic focus. The seizures originated from one hemisphere, but the retinal glial activation occurred both ipsi- and contralaterally. Furthermore, we show that the seizure-induced retinal glial activation can be significantly reduced with intracerebroventricular infusion of the same CX3CR1Ab as we have previously demonstrated to reduce microglial activation within the epileptic focus. This suggests common immunological features in the retina and the epileptic focus.

Although the retina exhibited a pronounced glial reaction, retinal neurodegeneration was subtle compared to the pathology within the epileptic focus [5, 17, 19, 23]. We found no evidence for prominent retinal neuronal death in terms of disseminated retinal lamination, increased numbers of pyknotic cells, or Fluoro-Jade-positive cells at the time points studied. Still, the increased number of activated microglia, often in clusters, disarranged the normally very strict territorial distribution of the retinal microglia [29], and is most likely reflecting alterations in their normal function as regulators of synaptogenesis and synaptic transmission [30, 31]. Similarly, the decreased levels of the excitatory synaptic scaffolding protein PSD-95 in the ONL, where we observed seizure-induced aberrant microglia processes, may indicate a homeostatic imbalance in excitatory transmission. The technique for intensity measurements was not sensitive enough for detecting minor differences in high intensity regions, which may explain why we did not detect changes in synaptic plexiform layers with strong PSD-95 expression [32]. Future electrophysiological recordings of retinal neuronal networks will be important for validating synaptic transmission and functional deficiencies.

Interestingly, other neurological diseases have also recently been described to induce retinal inflammation but with more pronounced neurodegeneration. In experimental and clinical studies on stroke, Parkinson’s, Huntingtons’, and Alzheimer’s diseases, reduced retinal ganglion cell numbers, and nerve fiber layer thinning are evident, causing visual disturbances [33]. Activation of micro- and macroglia, mononuclear infiltrations, and microvascular abnormalities have also been reported [33–39]. In addition, demyelinating diseases like multiple sclerosis show optic disc and nerve dysfunction [33]. Notably, experimental AD studies linked increased retinal glial activation to astrocyte changes in the brain [37]. However, since the presented delayed upregulation of GFAP in macroglia after SE is an early indicator of reactive gliosis [40], we cannot rule out that the pathology may progress beyond 7 weeks post-SE and with additional seizures. Conversely, since there was a high variation in retinal GFAP expression within both the SE and NSC group 6 h and 1 week after SE, we cannot rule out that there may be changes occurring acutely that were not detected with our analyses. The significance of the small decrease in GFAP at 6 h after SE also remains to be clarified.

Another important distinction between the current and previous studies on stroke and neurodegenerative diseases is the fact that the temporal seizures were initiated from implanted electrodes within a very localized area of an initially healthy brain, without genetic susceptibility or systemic immunological/vascular deficiencies. Hence, the retinal changes could only have occurred as a result of the SE insult, the SE-induced spontaneous seizures, and/or SE-induced epileptogenesis and not due to an underlying disease, which is otherwise often the case in epilepsy. Future correlation studies of seizure numbers and degree of retinal inflammation as well as studies on retinal inflammation in other models of epilepsy may give more answers to what extent retinal inflammation is a biomarker of epileptogenesis or merely a reaction to seizures per se.

There are at least four possible scenarios that may explain the underlying mechanisms of the seizure-induced retinal immune response. First, temporal seizures initiate an acute systemic immune response which may spread through the BRB. Common inflammatory mediators are upregulated in both the hippocampus and enthorinal cortex 24 h after SE [41]. However, when evaluating the same immune factors at an earlier time point in eye homogenates, no such increase was found. Instead, subtle changes in cytokine/chemokine levels were observed with a delay. A systemic immune response may also trigger a delayed infiltration of leukocytes from the retinal vessels in the subretinal or retinal layers. Though, we did not observe such an adaptive response, it may still occur at other time points. We also did not find changes in numbers of vascular pericytes, as in, i.e., diabetic retinopathy [42, 43]. Vascular pericytes are situated along endothelial cells in the wall of small blood vessels [44, 45] and sense microvascular dysfunction [46, 47]. Here, we cannot rule out that seizures lead to altered BRB function and a more subtle microvascular reorganization. Moreover, there are studies describing retinal microglial activation without prominent infiltrating leukocytes during a systemic inflammation and viral infections [14].

Secondly, the seizures may change the intracranial pressure (ICP) and influence the intraocular pressure (IOP) within the eyes. An imbalance between the anterior IOP and posterior ICP fluid pressures on the optic nerve may lead to abnormal function and nerve damage in GCL and ONL due to changes in axonal transport or altered blood flow, as seen in glaucoma [48, 49]. Initial signs of increased IOP may include altered PSD-95 expression and gliosis [50, 51].

Thirdly, the immune response in the epileptic focus may spread to other brain areas involved in the temporal seizure networks, including subcortical structures, and via the visual pathways initiate changes as far as the retina. It is likely that such a scenario would induce a stronger immune response close to the entrance of the optic nerve and inner part of the retina. Accordingly, we found macroglia activation primarily in the inner retina. However, the microglial cells did not gather within the inner layers but were evenly distributed within synaptic layers and aberrantly in the ONL. Detailed investigations of the nerve fiber tract and optic disc may reveal more subtle local changes in favor of a direct rostral cerebrum-to-retina signaling. In support, studies in AD patients suggest that inflammation may, through soluble pathogenic factors such as small nucleic acids, spread from the limbic structures both into the visual cortex, optic nerve, and the retina [39, 52].

Fourthly, an epileptic seizure may induce a retrograde current through the optic nerve, initiating an imbalance in excitatory/inhibitory transmission in the retinal network and thereby a glial activation. Whether the retinal neurons may have the properties to exhibit abnormal synchronized firing similar to a seizure among cortical neurons is to our knowledge not known. Activated Müller cell processes in the inner retina may perhaps contribute to epileptic discharges, as suggested for astrocytes in the rest of the brain [10].

The seizure-induced glial activation was evident in the retina both ipsi- and contralateral to the temporal epileptic focus. A previous case report on Rasmussen’s encephalitis, a severe inflammatory epileptic encephalopathy engaging only one brain hemisphere, described an ipsilateral ocular inflammation in the patient [53]. The authors speculated to what extent an immune reaction in the eye after focal seizures may also be associated with clinical manifestations. However, apart from small alterations in cytokine/chemokine levels in the ipsilateral retina only, we could not find significant differences between the two retinas after focal temporal seizures helping in determining whether the rat exhibited left or right temporal lobe seizures. In the current study, we did not differentiate between lateral and medial parts of the retina; and therefore, we cannot define whether the retinal inflammation may be correlated to the left or right hemisphere-innervated visual field. This will be important to analyze in future studies.

A reduced glial activation both in the retina and within epileptic foci following intracerebroventricular CX3CR1 Ab treatment suggests that the two immune responses, despite being remotely located, share similar activated signaling pathways. The fractalkine-CX3CR1 pathway is involved in the activation and migration of immune cells [27, 54, 55], and an increased expression of CX3CR1 in the epileptic focus suggests a dysfunctional neuron-glial cross talk [19, 56, 57]. Thus, a reduced immune response in the eye after CX3CR1 Ab treatment may be due to a reduced propagation of the immune response from the epileptic focus/network, which could be a result of both a reduced microglia activation and neurodegeneration within the epileptic focus and a direct inhibition of the migratory capacity of the immune cells. However, CX3CR1 deficiency has been associated with eye-related disorders [58–60]; thus, we do not know the functional consequences of inhibiting seizure-induced retinal immune response with CX3CR1 Ab. During healthy conditions, the glial cells are important for keeping the homeostasis. The seizure-induced retinal immune response or the remaining immune response after CX3CR1 Ab treatment may be dysfunctional. As for the immune response in the rest of the brain, the retinal immune response is likely to both contribute to and restore pathology [61]. However, we believe that the mere existence of a retinal glial activation may turn out to be a potential novel biomarker of seizure-induced or epileptogenesis-associated brain inflammation. The fact that the retina is more accessible than many other parts of the brain makes it more attractive for diagnostic purposes. Studying the immune reaction in the eyes of epilepsy patients and correlating it to the seizure semiology, development, and prognosis may turn out to be clinically relevant.

To what extent a visual dysfunction in patients with temporal epilepsy exists and correlates to a glial activation in the retina and brain remains to be shown. Patients with epilepsy do not commonly describe visual disturbances, except for those with visual cortical involvements or drug-induced side effects. Therefore, we do not currently know if patients have retinal inflammation and visual disturbances subclinically. In the present study, we have also not addressed the question as to what extent rodents with temporal epileptic seizures suffer from functional retinal deficiencies. Future studies with pattern evoked electroretinography and multi-electrode array analysis of retinal physiology will be highly relevant [62, 63].

## Conclusions

These are the first evidence that epileptic seizures lead to an immune response in the retina. The finding has a potential to become a novel non-invasive tool for detecting brain inflammation through the eyes.

## Abbreviations

Ab, antibody; AD, Alzheimer’s disease; BRB, blood-retina barriers; DAPI, 496-diamidino-2-phenylindole; EEG, electroencephalographic; ELISA, enzyme-linked immunosorbent assay; GAPDH, glyceraldehyde 3-phosphate dehydrogenase; GCL, ganglion cell layer; GFAP, glial fibrially acidic protein; Htx-eosin, hematoxylin-eosin; ICP, intracranial pressure; IFN-γ, interferon gamma; IL-1β, 4–6, 10, 13, interleukin 1beta, 4-6, 10, 13; IML, inner limiting membrane; INL, inner nuclear layer; IOP, intraocular pressure; IPL, inner plexiform layer; KC/GRO, keratinocyte chemoattractant/growth-related oncogene; MSD, Meso-Scale Discovery; NFL, nerve fiber layer; NG2, neuron glial antigen 2; NSC, non-stimulated control; ONL, outer nuclear layer; OPL, outer plexiform layer; PBS, phosphate-buffered saline; PFA, paraformaldehyde; PSD-95, postsynaptic density-95; SE, status epilepticus; TNF-α, tumor necrosis factor alpha
